# From past to present: opportunities and trends in the molecular detection and diagnosis of* Strongyloides stercoralis*

**DOI:** 10.1186/s13071-023-05763-8

**Published:** 2023-04-11

**Authors:** Abigail Hui En Chan, Urusa Thaenkham

**Affiliations:** Department of Helminthology, Mahidol University, Bangkok, Thailand

**Keywords:** Molecular detection, Molecular diagnosis, Soil-transmitted helminth, *Strongyloides stercoralis*, Strongyloidiasis

## Abstract

*Strongyloides stercoralis* is a soil-transmitted helminth that is mainly found in the tropical and subtropical regions and affects approximately 600 million people globally. The medical importance of strongyloidiasis lies in its capacity to remain asymptomatic and chronically unnoticed until the host is immunocompromised. Additionally, in severe strongyloidiasis, hyperinfection syndrome and larva dissemination to various organs can occur. Parasitological techniques such as Baermann-Moraes and agar plate culture to detect larvae in stool samples are the current gold standard. However, the sensitivity might be inadequate, especially with reduced worm burden. Complementing parasitological techniques, immunological techniques including immunoblot and immunosorbent assays are employed, with higher sensitivity. However, cross-reactivity to other parasites may occur, hampering the assay’s specificity. Recently, advances in molecular techniques such as polymerase chain reaction and next-generation sequencing technology have provided the opportunity to detect parasite DNA in stool, blood, and environmental samples. Molecular techniques, known for their high sensitivity and specificity, have the potential to circumvent some of the challenges associated with chronicity and intermittent larval output for increased detection. Here, as *S. stercoralis* was recently included by the World Health Organization as another soil-transmitted helminth targeted for control from 2021 to 2030, we aimed to present a review of the current molecular techniques for detecting and diagnosing *S. stercoralis* in a bid to consolidate the molecular studies that have been performed. Upcoming molecular trends, especially next-generation sequencing technologies, are also discussed to increase the awareness of its potential for diagnosis and detection. Improved and novel detection methods can aid in making accurate and informed choices, especially in this era where infectious and non-infectious diseases are increasingly commonplace.

**Graphical Abstract:**

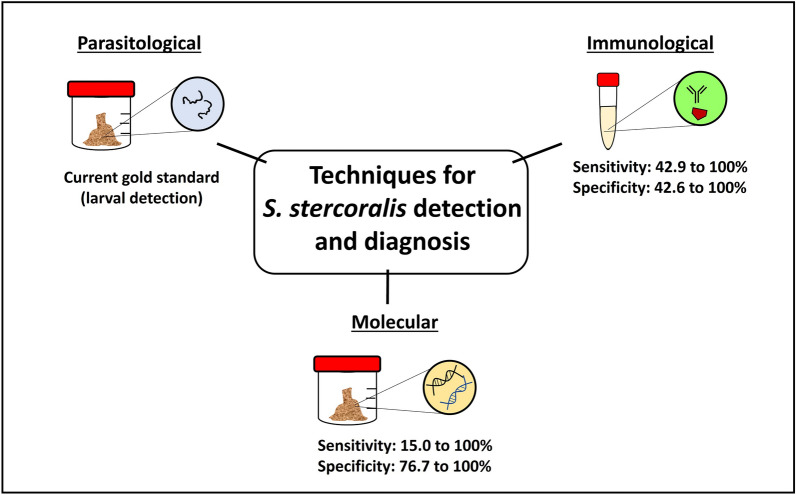

## Background

*Strongyloides stercoralis*, a soil-transmitted helminth (STH), is responsible for human strongyloidiasis, which is estimated to affect approximately 600 million people globally [1–3]. Strongyloidiasis is endemic in tropical and subtropical regions, and foci of infections have also been found in temperate countries, including Japan, Australia, and Italy [4]. *Strongyloides stercoralis* infection in humans ranges from asymptomatic light infections to chronic symptomatic infections. Severe strongyloidiasis can occur as hyperinfection syndrome (increased parasite burden resulting in high parasite load) and/or disseminated strongyloidiasis (presence of larva in other ograns aside from the gastrointestinal tract). Like a silent assassin, *S. stercoralis* infection can remain asymptomatic and chronically unnoticed until the host is immunocompromised [5, 6]. Hyperinfection is potentially life-threatening, with mortality rates of up to 85% in immunocompromised patients [7, 8]. Moreover, the unique ability of *S. stercoralis* to replicate itself in the human host allows for cycles of autoinfection, where the larva attains infectivity without leaving the host [9].

Currently, there is consensus regarding the underestimation of the actual prevalence rate of *S. stercoralis*, partly due to asymptomatic infections and inadequately sensitive methods for detection and diagnosis [3]. In contrast to other STHs where the gold standard of diagnosis is the presence of eggs in microscopic stool examination, *S. stercoralis* larvae are usually released in stool samples instead. Moreover, in asymptomatic infections where the larval output is low and intermittent, the sensitivity of stool examination may be compromised [10]. Other methods for *S. stercoralis* detection include immunological and molecular methods, which have been dubbed a more sensitive alternative to complement diagnosis. The current molecular methods include conventional polymerase chain reaction (PCR) and quantitative PCR (qPCR), which are widely used for the molecular detection and identification of parasitic helminths [10–12]. However, the effectiveness of PCR as a diagnostic tool for *S. stercoralis* diagnosis and detection remains subjective because of the differing sensitivities reported.

Recently, the World Health Organization (WHO) included *S. stercoralis* with the other STHs targeted for control from 2021 to 2030 [11]. Incorporating *S. stercoralis* into a WHO control program includes gaining knowledge of the epidemiology of *S. stercoralis*, conducting field evaluations and pilot projects, and finding a suitable standard diagnostic tool for detection and diagnosis [11]. Due to the inclusion of *S. stercoralis* as a target for control, the importance of a sensitive and accurate technique for molecular diagnosis is crucial.

In this study, to consolidate the molecular studies that have been conducted and to assist stakeholders in the WHO’s direction, we present an up-to-date review of the current molecular techniques used for detection and diagnosis of *S. stercoralis*. Additionally, upcoming molecular trends, especially next-generation sequencing technologies, are discussed in this context to increase awareness of their potential for diagnosis and detection.

## Techniques for *Strongyloides stercoralis* detection

### Parasitological techniques

Currently, parasitological techniques are the gold standard for detecting *S. stercoralis* larvae in fecal samples under microscopes [13]. Compared to other STHs, where eggs can be detected in fecal samples, *S. stercoralis* eggs are not usually found; thus, parasitological techniques like the simple smear or Kato-Katz are not suitable. More appropriate parasitological methods for larval detection include the Baermann-Mores and agar plate culture (APC) [14–17]. The sensitivity of the technique is crucial to make a correct diagnosis, as the failure to detect *S. stercoralis* does not indicate the unequivocal absence of infection [9]. Also, multiple fecal examinations have been proven to be more sensitive than a single examination [9, 18]. Knopp et al. (2008) revealed an increase in sensitivity from 6.3% (for single examination) to 10.8% (for multiple examinations) for *S. stercoralis* detection in a combination of Baermann-Moraes and APC [18]. Modifications in APC have also aided in improving the sensitivity and reducing bacterial contamination [19]. However, these methods are time-consuming and require trained parasitologists for detection and identification. Also, in cases where there is light infection and the larval output is intermittent and low, the sensitivity of parasitological techniques can be compromised.

Despite the low sensitivity, parasitological techniques remain the go-to method for *S. stercoralis* detection and diagnosis. They are commonly used as a benchmark to compare the efficacy of immunological and molecular techniques [20, 21]. Although there is a shift towards adopting combinations of various parasitological methods and immunological or molecular techniques, its specificity, low cost, and no requirement for special equipment allow for the ease of use, especially in field settings.

### Immunological techniques

Immunological techniques, such as enzyme-linked immunosorbent assay (ELISA), immunofluorescence antibody test (IFAT), and western blot, have been used as alternatives for *S. stercoralis* diagnosis and present certain advantages over parasitological methods [22]. Various studies have shown their high sensitivity, depending on the type of test employed [9, 22, 23]. Table 1 summarizes the sensitivity and specificity of the different immunological tests for the diagnosis of human strongyloidiasis. Among the 32 studies, the sensitivity ranged from 42.9% to 100%, while the specificity ranged from 42.6% to 100%.

**Table 1:** Summary of the studies on the sensitivity and specificity of immunological methods for strongyloidiasis

Immunological method^a^	Population sample	Sensitivity (%)	Specificity (%)	Reference method	References
ELISA IgG IVD commercial kit	Serum from pregnant women in the Peruvian Amazon	63.3	69.6	Parasitological	[58]
	Serum from Center for Tropical Diseases in Italy and National Institute of Health in the USA	91.2	99.1	Parasitological	[24]
	Serum from Universiti Sains Malaysia in Malaysia	84.6	83.6	Parasitological	[59]
ELISA IgG Bordier commercial kit	Serum from outpatients at Hospital for Tropical Diseases in London	81	NA	Parasitological	[60]
	Serum from patients at Rennes University Hospital in France	100	97	Parasitological	[29]
	Serum from Center for Tropical Diseases in Italy and National Institute of Health in the USA	89.5	98.3	Parasitological	[24]
ELISA crude antigen SciMedx commercial kit	Serum from patients in the Hospital Universitario 12 de Octubre in Spain	89.2 to 94.7	72.3 to 89.3	Composite reference and parasitological	[61]
ELISA IgG InBios Strongy Detect commercial kit	Serum submitted to laboratories in the USA	80	90	Parasitological	[62]
NIE-ELISA NovaLisa commercial kit	Serum from patients in the Hospital Universitario 12 de Octubre in Spain	72.3 to 78.9	85.1 to 93.6	Composite reference and parasitological	[61]
ELISA IgG Strongy Detect (both Ss-NIE and Ss-IR recombinant antigens) commercial kit	Serum from patients at the National Institute of Allergy and Infectious Diseases in the USA	98.6	98.6	Parasitological	[31]
ELISA IgG4 Strongy Detect (both Ss-NIE and Ss-IR recombinant antigens) commercial kit	Serum from patients at the National Institute of Allergy and Infectious Diseases in the USA	95.9	100	Parasitological	[31]
ELISA IgG (crude *Strongyloides ratti* antigen)	Urine from communities in northeast Thailand	83 to 85	53 to 56	Parasitological	[63]
	Serum from communities in northeast Thailand	100	42.6	Parasitological	[63]
	Serum from communities in northeast Thailand	84.5	100	Parasitological	[64]
ELISA IgG (crude *Strongyloides venezuelensis* antigen)	Serum from Hospital das Clinicas da Faculdade in Brazil	95	97.83	Parasitological	[65]
	Serum from Hospital das Clinicas da Faculdade in Brazil	92.5	93.48	Parasitological	[65]
	Serum from Instituto de Medicina Tropical Alexander von Humboldt in Peru	74.1	100	Parasitological	[66]
ELISA IgG (crude *Strongyloides stercoralis* antigen)	Serum from communities in northeast Thailand	73	86	Parasitological	[67]
	Serum from communities in northeast Thailand	83.5	100	Parasitological	[64]
	Serum from patients with hematologic malignancy at the University Hospital in Brazil	68.0	89.0	Parasitological	[68]
	Serum from Universiti Sains Malaysia in Malaysia	84.6	81.8	Parasitological	[59]
	Serum from patients with corticosteroid therapy in primary health care centers in Egypt	42.1	82.6	Parasitological	[69]
	Serum from Instituto de Investigacionces de Enfermedades Tropicales in Argentina	97	100	Parasitological	[70]
	Serum from immunocompromised patients in Phramongkutlao Hospital in Thailand	42.9	96.3	Parasitological	[27]
Centers for Disease Control and prevention EIA IgG (crude *Strongyloides stercoralis* antigen)	Serum from patients at Toronto General Hospital in Canada	94.6	NA	Parasitological	[71]
ELISA IgG	Serum from travelers attending the Hospital for Tropical Diseases in London	73	NA	Parasitological	[72]
ELISA IgG	Serum from immigrants attending the Hospital for Tropical Diseases in London	98	NA	Parasitological	[72]
ELISA IgG4 (crude *Strongyloides stercoralis* antigen)	Serum from Universiti Sains Malaysia in Malaysia	76.9	92.7	Parasitological	[59]
ELISA IgE (crude *Strongyloides stercoralis* antigen)	Serum from Universiti Sains Malaysia in Malaysia	100	100	Parasitological, molecular, immunological	[73]
	Serum from Universiti Sains Malaysia in Malaysia	7.7	100	Parasitological	[59]
ELISA IgY (crude *Strongyloides venezuelensis* antigen from larva)	Serum from Biological Samples Bank of Laboratório de Parasitologia in Brazil	95.56	88.89	Parasitological	[74]
ELISA IgY (crude *Strongyloides venezuelensis* antigen from adult females)	Serum from Biological Samples Bank of Laboratório de Parasitologia in Brazil	95.56	91.11	Parasitological	[74]
ELISA IgG (synthetic peptide C10)	Serum from patients	95	89.2	Parasitological	[75]
ELISA IgG (synthetic peptide D3)	Serum from patients	95	92.5	Parasitological	[75]
ELISA IgG4 (*Strongyloides stercoralis* rSs1a recombinant antigen)	Serum from Universiti Sains Malaysia in Malaysia	96	93	Parasitological, molecular, immunological	[76]
ELISA IgG (SsAg recombinant monoclonal antibody)	Serum bank at University Sains Malaysia in Malaysia	100	100	Parasitological and immunological	[77]
ELISA (*Strongyloides stercoralis* recombinant 14-3-3 protein)	Serum from patients	96	NA	Parasitology	[78]
NIE-ELISA	Serum from Center for Tropical Diseases in Italy and National Institute of Health in the USA	75.4	94.8	Parasitological	[24]
	Serum from Instituto de Investigacionces de Enfermedades Tropicales in Argentina	84	100	Parasitological	[70]
	Serum from communities in Argentina	76.7	71.6	Bayesian latent class analysis estimates	[79]
	Dried blood spots from indigenous community in Australia	85.7	88.9	Parasitological	[80]
NIE-LIPS	Serum submitted to laboratories in the USA	100	100	Parasitological	[62]
	Serum from Center for Tropical Diseases in Italy and National Institute of Health in the USA	85.1	100	Parasitological	[24]
	Serum from Instituto de Investigacionces de Enfermedades Tropicales in Argentina	97.8	100	Parasitological	[70]
NIE dot-based assay	Serum from multiple reference laboratories	96.3	100	Parasitological	[81]
SsIR-LIPS	Serum from Instituto de Investigacionces de Enfermedades Tropicales in Argentina	91.2	100	Parasitological	[70]
IFAT (*Strongyloides stercoralis* larva)	Serum from patients at the Centre for Tropical Diseases in Italy	95.5	NA	Composite reference	[16]
	Serum from Center for Tropical Diseases in Italy and National Institute of Health in the USA	93.9	92.2	Parasitological	[24]
Gelatin particle indirect agglutination assay (crude *Strongyloides stercoralis* antigen)	Serum from communities in northeast Thailand	81	81	Parasitological	[67]
	Serum from patients with corticosteroid therapy in primary health care centers in Egypt	89.4	81.8	Parasitological	[69]
Gelatin particle indirect agglutination (crude *Strongyloides venezuelensis* antigen)	Serum from Instituto de Medicina Tropical Alexander von Humboldt in Peru	98.2	100	Parasitological	[66]
ICT (crude *Strongyloides stercoralis* antigen)	Serum from Khon Kaen University in Thailand	93.3	83.7	Parasitological	[32]
Lateral flow rapid dipstick test IgG4 (SsRapid^™^)	Serum from northeast Thailand	82	96	Parasitological, immunological	[82]
	Serum from Universiti Sains Malaysia in Malaysia	91.3	100	Parasitological, molecular, immunological	[33]

^a^*EIA* enzyme immunosorbent assay, *ELISA* enzyme-linked immunosorbent assay, *ICT* immunochromatographic test, *IFAT* immunofluorescene antibody test, *LIPS* luciferase immunoprecipitation systems assay

The sensitivity of five immunological tests (consisting of in-house assays and commercially available ELISA tests) was compared by Bisoffi et al. (2014), and their results revealed that the sensitivity among the tests ranged from 75.4% to 93.9%, with the IFAT test being the most sensitive [24]. However, studies have also revealed cross-reactivity with other helminthic infections, such as filariasis and schistosomiasis, when crude antigens are used [5, 22, 25]. Also, immunological tests cannot distinguish between current and past infections of *S. stercoralis*, which can be a limiting factor in areas where strongyloidiasis is endemic [23, 26]. Moreover, the sensitivity of immunodiagnostics can be reduced in cases where the host is severely immunosuppressed. In a study performed on immunocompromised patients in Thailand, the sensitivity was reported to be 42.9% using IgG indirect ELISA [27]. Currently, newer and more convenient immunodiagnostic tests are being developed to increase the specificity and reduce the time taken for results. These include the development of a commercial ELISA and a luciferase immunoprecipitation system using recombinant antigens (LIPS-NIE) that have no cross-reactivity with other STHs [24, 28–30]. Recently, a commercial ELISA kit (Strongy Detect, Inbios) with both recombinant antigens Ss-NIE and Ss-IR showed high sensitivity and specificity for IgG and IgG4 [31]. In addition, rapid tests like point-of-care cassettes and dipstick tests have been developed to rapidly detect strongyloidiasis [32, 33]. In recent years, a combination of parasitological and immunological techniques has been used for diagnosis and has proven to be more robust than parasitological techniques alone [10]. Although immunological techniques, with their high sensitivity, present a suitable complement to parasitological techniques, their low specificity and sensitivity, especially in immunocompromised hosts, remain a current limitation.

### Molecular techniques

Molecular techniques have been touted as a promising tool for *S. stercoralis* diagnosis and identification, with their potential for increased sensitivity and specificity [12, 20]. Table 2 summarizes the molecular-based studies conducted with their sensitivity and specificity values for *S. stercoralis* detection. Of the 24 studies reviewed, the sensitivity ranged from 15 to 100%, while specificity ranged from 76.7% to 100%, with different studies utilizing parasitological or immunological techniques, or both as references. The majority of studies conducted used fecal samples, while three studies used urine samples for the detection of *S. stercoralis* DNA. The most common genetic marker used was the nuclear 18S ribosomal RNA (rRNA) gene, with 16 out of 24 (66%) studies using the 18S primers and assay developed by Verweij et al. (2009) [34].

**Table 2:** Summary of studies on the sensitivity and specificity of molecular techniques for *Strongyloides stercoralis* detection and diagnosis

Genetic marker	Primer used	Type of PCR^a^	Sample type	Sensitivity (%)	Specificity (%)	Reference method	References
18S	[34]	RT-PCR	Fecal	72 to 92	100	Parasitological	[34]^b^
				84.7	95.8	Parasitological	[83]
				76.8	89.7	Parasitological	[84]
				15 to 34.1	> 99	Parasitological	[87]
				93.8	86.5	Parasitological	[35]
				90	85.7	Parasitological	[36]
				27.5 to 86.3	NA	Parasitological	[45]
				73.9	100	Parasitological	[39]
				85	87.3	Parasitological	[25]
				38	100	Immunological	[85]
				63	NA	Immunological	[20]
				57	NA	Parasitological and immunological (composite reference)	[16]
		Multiplex RT-PCR	Fecal	17.4 to 76.3	93.9	Parasitological and molecular combination	[15]
				88.9	92.7	Parasitological	[86]
		cPCR	Fecal	100	NA	Parasitological	[21]
				76.7	84.3	Parasitological	[25]
				78.8 to 84.8	82.5 to 95	Parasitological	[87]
				100	NA	Parasitological	[88]
	[89]	cPCR	Fecal	100	NA	Parasitological	[89]^b^
		Nested PCR	Fecal	75	NA	Parasitological	[89]^b^
	[37]	Multiplex RT-PCR	Fecal	72 to 100	100	Parasitological	[37]^b^
	[38]	RT-PCR	Fecal	82	76.7	Parasitological	[38]^b^
		ddPCR	Fecal	98	90	Parasitological	[38]^b^
*COI*	[90]	Nested PCR	Fecal	100	91.6	Parasitological	[90]^b^
ITS2	[89]	cPCR	Fecal	61	NA	Parasitological	[91]
	[92]	cPCR	Fecal	100	NA	Parasitological	[92]^b^
Repetitive elements	[46]	cPCR	Urine	93.6	NA	Parasitological	[46]^b^
				17	NA	Immunological	[20]
				74.7	77.1	Bayesian estimates	[79]

^a^*RT-PCR* real-time PCR, *cPCR* conventional PCR, *ddPCR* droplet digital PCR

^b^The reference indicates that the primers were originally developed in that particular study

The assay by Verweij et al. (2009) [34] targets the nuclear 18S rRNA gene using a real-time PCR (RT-PCR) assay for the detection of *S. stercoralis* in fecal samples [34]. Since its development, the assay and primers have been widely adopted by the scientific community, for both conventional and RT-PCR [21, 35, 36]. Also, multiplex PCR has been developed to simultaneously detect other STHs along with *S. stercoralis*, enhancing the utility of molecular techniques for diagnostics and detection [37]. Aside from the 18S rRNA gene primers by Verweij et al. (2009), other primers targeting the 18S rRNA gene and different PCR techniques have been employed. Of note, Iamrod et al. (2021) [38] developed and tested a droplet digital PCR (ddPCR) assay for *S. stercoralis* detection in fecal samples [38]. The study revealed higher sensitivity and specificity using ddPCR compared to RT-PCR and parasitological techniques. Although other genetic markers like the mitochondrial cytochrome c oxidase subunit I (*COI*) gene, internal transcribed spacer 2 (ITS2) region, and repetitive units have been used, the 18S rRNA gene remains a popular choice for *S. stercoralis* detection.

Although the sensitivity range of molecular techniques varies greatly (from 15 to 100%), molecular techniques are still highly valuable as a diagnostic tool, as only five studies reported a sensitivity of < 50%. In a systematic meta-analysis of molecular diagnostic accuracy for *S. stercoralis*, the accuracy was estimated to be 71.76% using parasitological techniques as the reference and 61.85% using either parasitological or immunological techniques [12]. The advantages of utilizing molecular techniques to diagnose *S. stercoralis* outweigh their limitations. First, molecular detection outperforms parasitological techniques such as spontaneous sedimentation in terms of sensitivity, and studies have revealed that the sensitivity and accuracy of diagnosis increase when a combination of techniques is applied in conjunction. Hailu et al. [39] tested five diagnostic methods (RT-PCR and four other parasitological methods) for *S. stercoralis* and revealed a higher detection rate when a combination of parasitological and molecular techniques was used as compared to a single diagnostic method [39]. The advantages and limitations of each of the three techniques for *S. stercoralis* detection are summarized in Table 3. Using a combination of techniques, the positivity rate increased from 10.9% (APC) or 28.8% (RT-PCR) to 36% when both APC and RT-PCR were employed. Second, DNA from dead larvae can be detected via PCR, while the larvae have to be alive for detection via APC or Baermann. Third, the simultaneous detection of other helminths and species identification can also be performed via molecular techniques, enhancing the efficiency. Finally, in terms of specificity, molecular techniques have the edge over immunological techniques. Although the sensitivity of molecular techniques is hindered by similar factors as parasitological techniques, such as low and intermittent larval output, these limitations can hopefully be overcome in the near future through the use of novel molecular methods with their increased sensitivity for detection.

**Table 3:** Advantages and limitations of each technique for *Strongyloides stercoralis* detection

Techniques	Advantage	Limitation
Parasitological	•Lower cost compared to immunological and molecular techniques •Easily implementable in a field setting	•Require increased sampling for higher sensitivity due to irregular larva output or asymptomatic patients •Possible misdiagnosis with hookworms due to similar morphology •Require live larva •Risk of *S. stercoralis* contamination when APC is used
Immunological	•Higher sensitivity than parasitological and molecular techniques •Not limited by the larval output •Able to detect other pathogens through multiplex assays •Possible to detect other biological materials such as breast milk and saliva	•Potential for cross-reactivity with other helminthiases •Persistence of antibodies renders the technique unable to distinguish between past and present infections (especially in endemic areas) •Lowered sensitivity for immunocompromised host
Molecular	•Higher sensitivity than parasitological techniques (direct examination, spontaneous sedimentation, or Kato-katz) •Higher specificity than serological techniques •Lower expertise is required than parasitological techniques •Ability to detect dead larva •Increased accuracy with molecular identification •Able to detect other pathogens through multiplex assays (Multiplex PCR) •Possible to detect from other environments, not only from stool, and urine samples	•Lack of standard for PCR and DNA extraction, causing varied sensitivity, and specificity •Require increased sampling for higher sensitivity due to irregular larva output or asymptomatic patients

## Current molecular trends and novel tools for *Strongyloides stercoralis* detection

Aside from diagnosis and detection, molecular techniques also allow the study of *S. stercoralis* molecular identification, phylogenetics, and genetic diversity. Other types of molecular-based studies performed for *S. stercoralis* are summarized in Table 4. These consist of cross-sectional, molecular identification, phylogenetics, genetic diversity, and molecular technique modification and improvement studies. Aside from fecal and urine samples, most studies have performed larval isolation of *S. stercoralis* prior to individual worm DNA extraction. Other types of sample include serum, cerebrospinal fluid (CSF), and bronchoalveolar lavage fluid to detect the presence of *S. stercoralis* DNA. The various types of genetic markers used include the nuclear 18S and 28S rRNA genes, ITS1 region, major sperm protein (MSP) gene, the mitochondrial *COI*, 12S and 16S rRNA genes, and repetitive elements. Although these genetic markers can be used for molecular identification and phylogenetic studies, the 18S rRNA and *COI* genes are highly popular. For the 18S rRNA gene, Hasegawa et al. (2009) [40] suggested the use of the hypervariable regions (named HVR-I, II, III, IV) to explore genetic differences between *S. stercoralis* populations [40]. With its high sequence variation, the mitochondrial *COI* gene is another genetic marker used to study the population genetics and diversity of *S. stercoralis* in different hosts and localities [41–43]. These genetic markers have proven helpful for the molecular identification of cryptic species and in aiding to shed light on the zoonotic potential of *S. stercoralis* through comparative molecular studies on dog and human isolates [42].

**Table 4:** Summary of molecular studies for *Strongyloides stercoralis*

Genetic marker	Type of PCR^a^	Sample type^b^	Type of study	References
18S	cPCR	Fecal	•Cross sectional	[93]
			•Molecular technique	[21]
			•Cross sectional	[94]
			•Prospective	[95]
			•Cross sectional	[96]
		Larva	•Case report	[97]
			•Cross sectional	[98]
			•Case report	[99]
			•Molecular technique	[40]
			•Cross sectional and phylogenetics	[100]
			•Cross sectional and phylogenetics	[101]
			•Cross sectional, phylogenetics, and genetic diversity	[42]
			•Cross sectional, phylogenetics, and genetic diversity	[102]
			•Phylogenetics	[103]
			•Cross sectional and genetic diversity	[104]
			•Case report	[105]
			•Cross sectional and phylogenetics	[106]
			•Cross sectional and phylogenetics	[107]
		Serum	•Cross sectional	[47]
	Multiplex cPCR	Larva	•Molecular technique	[108]
	RT-PCR	Fecal	•Cross sectional	[109]
			•Cross sectional	[110]
			•Cross sectional	[111]
			•Cross sectional	[112]
			•Cross sectional	[113]
	Multiplex RT-PCR	Larva	•Molecular technique	[114]
	LAMP	Urine	•Molecular technique	[52]
	cPCR, Illumina	Fecal	•Cross sectional, phylogenetics, molecular technique	[115]
	cPCR, RT-PCR, Illumina	Fecal	•Cross sectional, phylogenetics, genotyping	[116]
28S	cPCR	Larva	•Cross sectional and phylogenetics	[101]
	RT-PCR	Fecal	•Molecular technique	[117]
	LAMP	Larva	•Molecular technique	[118]
ITS1	Nested PCR	Fecal	•Cross sectional	[119]
		Larva	•Cross sectional	[120]
	Multiplex cPCR	Fecal	•Case report	[121]
Repetitive elements	RT-PCR, Illumina	Larva	•Molecular technique	[122]
MSP	cPCR	Larva	•Cross sectional and phylogenetics	[101]
12S	cPCR	Larva	•Phylogenetics	[123]
	Illumina	Larva	•DNA metabarcoding	[51]
16S	cPCR	Larva	•Phylogenetics	[123]
	Illumina	Larva	•DNA metabarcoding	[51]
*COI*	cPCR	Larva	•Cross sectional and phylogenetics	[100]
			•Case report	[99]
			•Cross sectional and phylogenetics	[101]
			•Cross sectional, phylogenetics, and genetic diversity	[42]
			•Cross sectional, phylogenetics, and genetic diversity	[102]
			•Phylogenetics	[124]
			•Case report	[105]
			•Cross sectional and phylogenetics	[106]
		Serum	•Cross sectional	[47]
	Nested PCR	Fecal	•Phylogenetics and genetic diversity	[41]
	cPCR, Illumina	Fecal	•Cross sectional, phylogenetics, molecular technique	[115]
			•Cross sectional, phylogenetics, genotyping	[116]
Metagenome	Illumina	Cerebrospinal fluid	•Case report	[50]
		Bronchoalveolar lavage fluid	•Cross sectional	[49]
			•Cross-sectional	[48]
Whole genome	Illumina	Larva	•Genomics	[125]
			•Cross sectional and phylogenetics	[106]
			•Phylogenetics and genomics	[126]

^a^*RT-PCR* real-time PCR, *cPCR* conventional PCR

^b^The fecal sample type indicates that molecular detection was performed directly from the fecal sample, while the larva sample type indicates that *Strongyloides* larvae were first isolated from the fecal sample and molecular identification was performed using the isolated larvae

Researchers have recently attempted to increase the diagnostic sensitivity for *S. stercoralis* detection. First, parasitological, immunological, and molecular techniques are increasingly employed for screening and confirmatory testing to broaden the net cast and to increase the detection accuracy rather than relying on one approach [16, 44]. Zueter et al. (2014) used fecal and serum samples collected from cancer patients to detect *S. stercoralis* through these three techniques [44]. Second, improvements have been made in the DNA extraction and PCR protocols for molecular detection via fecal samples. Examples include the removal of PCR inhibitors in fecal samples, enhancing DNA extraction methods, and exploring different sample types, such as urine and other bodily fluids, to determine if they can be used for diagnostics [8, 45, 46]. Cell-free DNA is also being explored, where molecular detection using the 18S rRNA and *COI* genes has been used to detect *S. stercoralis* in serum samples [47]. Third, the increasing trend in the use of next-generation sequencing (NGS) technologies for molecular-based studies is slowly gaining traction for helminth diagnostics. Illumina sequencing metagenomics were used to detect *S. stercoralis* in CSF and bronchoalveolar lavage fluid samples from patients, showing the high sensitivity of the technique and potential for use [48–50]. Additionally, targeted amplicon Illumina sequencing of the 12S and 16S rRNA genes through DNA metabarcoding has also demonstrated the potential of detecting *S. stercoralis* larvae spiked in mock helminth communities and environment matrices [51]. Although conventional molecular-based methods are still popular, the shift toward NGS is certain in the future. The use of NGS compared to conventional molecular-based methods can be highly advantageous because of their high sensitivity, decreased cost, and increased convenience.

In addition to increasing the sensitivity of *S. stercoralis* detection, the convenience of molecular detection in the field is another advantage. A loop-mediated isothermal amplification (LAMP) assay was successfully developed by Fernández-Soto et al. (2020) [52] using human urine and fecal samples for *S. stercoralis* detection [52]. Another interesting concept is the use of portable systems such as the portable Bento Lab, which is fully equipped with DNA extraction, PCR, and sequencing devices suitable for use in the field. Using the Bento Lab and the MinIon sequencer, DNA barcoding of parasitic and free-living nematode species was successfully performed directly in the field setting and was identified with 96 to 100% accuracy [53]. Lastly, as strongyloidiasis can be positively associated with hosts with underlying disease conditions, concurrent screening for strongyloidiasis and other diseases should be undertaken, especially for immunocompromised patients or patients requiring immunosuppressive drugs. Co-infection of strongyloidiasis with COVID-19 has been reported as well as *Strongyloides* hyperinfection syndrome resulting from treatment with corticosteroids for COVID-19 [54–56]. With infectious diseases being commonplace, there is an increasing need to screen for *Strongyloides* to prevent potentially fatal scenarios, especially when the use of corticosteroids is evident [57].

## Conclusions

The application of molecular techniques is undoubtedly vital to determine the true prevalence and disease burden of *S. stercoralis*. As each technique (parasitological, immunological, and molecular) has its benefits and drawbacks, none should be used as a stand-alone test for diagnosis. Molecular techniques can play a confirmatory role in diagnosis, with their ability to circumvent both the low sensitivity of parasitological techniques and the low specificity of immunological techniques. With molecular techniques advancing at an extraordinary pace, it is certainly a keystone in strongyloidiasis detection, especially in an era where infectious diseases and zoonoses are increasing in frequency.

## Data Availability

All data generated or analyzed during this study are included in this published article.

## Abbreviations

APC: Agar plate culture
*COI*: Cytochrome c oxidase subunit I
cPCR: Conventional PCR
CSF: Cerebrospinal fluid
ddPCR: Droplet digital polymerase chain reaction
ELISA: Enzyme-linked immunosorbent assay
IFAT: Immunofluorescence antibody test
ITS: Internal transcribed spacer
LAMP: Loop-mediated isothermal amplification
LIPS-NIE: Luciferase immunoprecipitation system using recombinant antigens
MSP: Major sperm protein
NGS: Next generation sequencing
PCR: Polymerase chain reaction
qPCR: Qualitative polymerase chain reaction
rRNA: Ribosomal RNA
RT-PCR: Real-time polymerase chain reaction
STH: Soil-transmitted helminth
WHO: World Health Organization
